# A Risk Assessment Matrix for Public Health Principles: The Case for E-Cigarettes

**DOI:** 10.3390/ijerph14040363

**Published:** 2017-03-31

**Authors:** Daniela Saitta, Azim Chowdhury, Giancarlo Antonio Ferro, Federico Giuseppe Nalis, Riccardo Polosa

**Affiliations:** 1 Department of Clinical and Experimental Medicine, University of Catania, Via Palermo 636, 95121 Catania, Italy; danisaitta@unict.it; 2 Keller and Heckman LLP, 1001 G Street N.W., Suite 500W, Washington, DC 20001, USA; chowdhury@khlaw.com; 3 Department of Law, University of Catania, Via Gallo 24, 95124 Catania, Italy; giancarloantonioferro@gmail.com; 4 School of Specialization in Occupational Medicine, University of Messina, Via Consolare Valeria 1, 98122 Messina, Italy; nalis@hotmail.it; 5 Centre for Tobacco Cessation and Prevention, Teaching Hospital “Policlinico-V. Emanuele”, Via S. Sofia 78, 95123 Catania, Italy

**Keywords:** cigarette smoking, electronic cigarettes, tobacco harm reduction, risk continuum, risk assessment, regulation, public health

## Abstract

Besides nicotine replacement therapies, a realistic alternative for smoking cessation or for smoking substitution may come from electronic cigarettes (ECs), whose popularity has been steadily growing. As for any emerging behaviour associated with exposure to inhalational agents, there is legitimate cause for concern and many health organizations and policy makers have pushed for restrictive policy measures ranging from complete bans to tight regulations of these products. Nonetheless, it is important to reframe these concerns in context of the well-known harm caused by cigarette smoking. In this article, we discuss key public health principles that should be considered when regulating ECs. These include the concept of tobacco harm reduction, importance of relative risk and risk continuum, renormalization of smoking, availability of low-risk product, proportionate taxation, and reassessment of the role of non-tobacco flavours. These public health principles may be systematically scrutinized using a risk assessment matrix that allows: (1) to determine the measure of certainty that a risk will occur; and (2) to estimate the impact of such a risk on public health. Consequently, the ultimate goal of responsible ECs regulation should be that of maximizing the favourable impact of these reduced-risk products whilst minimizing further any potential risks. Consumer perspectives, sound EC research, continuous post-marketing surveillance and reasonable safety and quality product standards should be at the very heart of future regulatory schemes that will address concerns while minimizing unintended consequences of ill-informed regulation.

## 1. Introduction

Electronic cigarettes (ECs) are electrically driven consumer products consisting of a battery part and a heating element (atomizer) that vaporizes a liquid (mainly consisting of propylene glycol, vegetable glycerine, distilled water, flavourings) that may or may not contain liquid nicotine. Vaporization allows for inhalation of vapour (referred to as “vaping”) and produces an aerosol similar in appearance but different in substance and chemistry to conventional cigarette smoke.

ECs come in a large variety of designs, shapes and sizes: some look like traditional cigarettes, cigars, or pipes, others look similar to a pen or small flashlights; some are single-use disposables, or use prefilled cartridges and others have refillable cartridges or tanks (e.g., “open-system” vaporizers).

The success of e-cigarettes appears in large part to be related to the fact that they share many similarities with smoking in the behavioural aspect of use [1,2]. As confirmed also by the data from clinical trials [3,4,5,6], users report using them long term to reduce cigarette consumption or quit smoking, to relieve tobacco withdrawal symptoms, and to continue having a “smoking experience without smoking” [7,8], with much reduced health risks [9]. Moreover, the fact that they can be used in many areas where smoking is prohibited, that they have a very competitive price, and are perceived as a much less harmful smoking alternative [10], increases their popularity.

In the face of the growing popularity of e-cigarettes among users, many public health experts, large health organizations and policy makers have expressed concerns for individual health risks, and concerns for population effects, that can be summarized as: whether e-cigarette (long-term) use results in higher overall absorption of nicotine/nicotine overdose, or some as-yet-unknown health risks associated with vapour constituents; whether e-cigarettes perpetuate smokers’ addiction to nicotine; whether the use of e-cigarettes serves as a gateway to smoking for non-users, particularly youth; whether e-cigarettes make smoking socially acceptable again, thus undermining decades of tobacco stigmatization efforts and current no-smoking policies.

There is now emerging evidence that could provide reassuring answers to these questions [11,12]. Nevertheless, international health organizations and regulatory authorities in many countries—by invoking a very constraining version of the precautionary principle—have hurriedly recommended and introduced restrictions on the manufacturing, import, sale and advertising of e-cigarettes. 

Essentially, three regulatory classifications have been applied, during the last decade, to ECs: general consumer product, medicinal product and tobacco product. All pros and cons have been appraised in a recent article that examines and summarizes these categories at the European Union level [13].

With regard to the consumer product regulation, in the European Union, the General Product Safety Directive 2001/95/EC (GPSD) regulates consumer products by subjecting them to general safety requirements, with regard to their characteristics (composition, packaging, labelling, and instructions), and to their effects on other products. According to the GPSD, a product may be declared safe when it conforms to relevant EU and national laws and/or, among other things, to voluntary national standards. The fact is that there is no EU-wide standard on e-cigarettes. A first attempt to create a voluntary national standard comes from the UK, where the British Standards Institute (BSI) and the Electronic Cigarette Industry Trade Association (ECITA) developed on July 2015 a Publicly Available Specification (PAS 54115) [14] giving guidance for the manufacture, import, labelling, marketing and sale of vaping products, including electronic cigarettes, e-shishas and DIY e-liquid mixing kits. Among other things, the standard covers: (i) purity of e-liquid ingredients, potential contaminants from device materials and potential emissions from device operation; (ii) a test solution-liquid, and an outline for the toxicological and chemical analysis of emissions; (iii) safety of batteries and chargers [13]. Even if the voluntary standard has not a mandatory value, the strategy of maintaining the general requirement that products must be safe, coupled with could be very useful to approach EC in a proportionate way, allowing for flexibility, innovation, affordability. 

In the United States, the Consumer Product Safety Commission (CPSC) has jurisdiction over a broad range of consumer products under a variety of statutes, including the Consumer Product Safety Act (CPSA) [15], the Federal Hazardous Substances Act (FHSA) [16] and the Poison Prevention Packaging Act (PPPA) [17]. Under the CPSA, it is unlawful to manufacture for sale, to distribute in commerce, or to import into the U.S. any consumer product that does not conform to an applicable product safety standard, or that is a banned hazardous substance under the FHSA (U.S. Code, Section 19(a)(1) of CPSA). Certain product categories are specifically exempted under CPSA including motor vehicles, pesticides, firearms, aircraft, boats, food, drugs, cosmetics, medical devices and tobacco (U.S. Code, Section 3(a)(5)(A)–(I) of CPSA). Despite the fact that e-cigarettes contain components and parts such as batteries that are typically regulated by the CPSC, the agency has not yet asserted any authority over e-cigarettes in the United States, likely because e-cigarettes that contain nicotine derived from tobacco are considered “tobacco products” under the Food, Drug and Cosmetic Act (FDCA), as amended by the Family Smoking Prevention and Tobacco Control Act of 2009 (Tobacco Control Act), as discussed below. Further, any e-cigarettes that are marketed with therapeutic claims are considered drugs or drug delivery devices, products that are also under FDA’s authority. However, considering that CPSC has traditionally taken an expansive view of its jurisdiction, the agency could potentially assert jurisdiction over certain elements of the e-cigarette electronic delivery mechanism if, for example, a consumer product safety issue arose because of overheating devices, batteries, or electronic failures. 

Of particular relevance for the U.S. refillable e-liquid industry is the Poison Prevention Packaging Act (PPPA), which is administered by the CPSC [18]. This law requires certain household products (e.g., certain chemical, cosmetic, drugs, dietary supplements and mouthwash), but not yet e-cigarettes or e-liquids, to be packaged in “child-resistant packaging”, which is packaging designed or constructed to be significantly difficult for children under five years of age to open within a reasonable time, and not difficult for normal adults to use properly. On 26 July 2016, the Children’s Nicotine Poisoning Prevention Act (CNPPA) became effective and requiring manufacturers of nicotine-containing e-liquids to comply with the child-resistant packaging and testing requirements set forth in the PPPA [19]. 

While it is possible to achieve a relatively high level of technical regulation via general consumer product rules, it must be said that the lack of a pre-market regulatory review, the gaps in the regulation of the quality of the manufacturing and supply chain, and the absence of a clear system for post-market monitoring and surveillance has led some legislators and regulators to decide to regulate e-cigarettes as medicines. 

In the U.S., medicinal or drug products are regulated by FDA’s Center for Drug Evaluation and Research (CDER). The term “drug” is defined in Section 201(g)(1) of the FDCA, in pertinent part, as “articles intended for use in the diagnosis, cure, mitigation, treatment or prevention of disease in man or other animals” and “articles (other than food) intended to affect the structure or any function of the body of man or other animals”. Therefore, whether an e-cigarette is considered a drug in the U.S. depends on its intended use. 

Regarding this, in January 2017 FDA published a new rule clarifying when a tobacco product is actually a medical product (drug) [20]. In that rule, reiterates that a product made or derived from tobacco will be subject to FDA’s medical product authority, and not its tobacco authority, (a) if it is intended for use in the diagnosis of disease or other conditions, or in the cure, mitigation, treatment or prevention of disease, including use in the cure or treatment of nicotine addiction (e.g., smoking cessation), relapse prevention, or relief of nicotine withdrawal symptoms; or (b) if it is intended to affect the structure or any function of the body in any way that is different from effects related to nicotine that were commonly and legally claimed in the marketing of cigarettes and smokeless tobacco products prior to 21 March 2000. 

When determining the intended use of a product, FDA will look primarily at the express claims being made by the manufacturer on the product’s labelling, website and marketing materials. Thus, if an e-cigarette company markets its products as having a therapeutic benefit, such as for smoking cessation or as a nicotine replacement therapy, those products would fall under FDA’s drug authority, even if the nicotine used in them is derived from tobacco. FDA’s new rule makes clear that any representation by a manufacturer in a public forum can provide evidence of intended use, including communications and reports to other administrative agencies or company websites [20]. Importantly, FDA may also look at how the product is actually being used by consumers as evidence of the manufacturer’s intent.

For e-cigarette companies, this means that FDA could look at the representations made by those affiliated with the product, as well as how the product is actually used by consumers, to determine whether a particular e-cigarette is intended for a therapeutic benefit (again in 21 C.F.R. § 201.128). If FDA finds that an e-cigarette or e-liquid is intended for such a therapeutic benefit, such product would be considered a new drug that can be marketed only after a new drug application (NDA) has been approved by FDA. Through the NDA process, drug manufacturers are charged with ensuring that their products are safe and effective for their intended use before they reach the market. E-cigarettes that contain nicotine derived from tobacco (or other tobacco derived substances), and are intended and marketed for recreational use, are considered instead tobacco products under the FDCA [21]. 

In the European Union, the same strategy has been applied by the UK government, where two separate routes exist under which ECs can be regulated: as tobacco products under the EU Tobacco Products Directive 2014/40/EU (TPD) [22] and as medical products with licensing from the Medicines & Healthcare products Regulatory Agency (MHRA). In 2013, pending the adoption of the TPD, the MHRA was encouraging companies voluntarily to license their nicotine-containing products (NCPs) irrespective of whether the manufacturer made any medicinal claims linked to smoking cessation or alleviation of the symptoms of nicotine withdrawal, with the aim to “ensure that products are available that meet appropriate standards of safety, quality and efficacy to help user cut down their smoking and to quit” [23]. With the adoption of the TPD, in practical terms, every nicotine-containing product marketed in the UK having a concentration of nicotine higher than the maximum level allowed by the TPD (20 mg/mL) must be regulated as a medicine. The license process as a medicine is probably the one with the highest standards for the protection of public health, but the extensive and costly pre- and post-authorization process has forced many little EC companies to opt for regulating their products under the TPD. Indeed, a nicotine inhaler, which closely resembles a cigarette, produced by a big tobacco company is the first product of its kind to be licensed as a medicine in the UK. As a medicinal product delivering a precise dose of nicotine, it can be prescribed by doctors for patients trying to quit smoking [24].

The final regulatory option is to regulate e-cigarettes as tobacco products. Although nicotine is usually manufactured from tobacco, pure nicotine does not contain tobacco per se, which is a feature of many legal definitions of tobacco products. Under such circumstances, bringing e-cigarettes within the scope of the tobacco rules may require legislation [13]. 

In the U.S., the Tobacco Control Act amended the FDCA to give FDA authority over the manufacture, labelling, marketing and distribution of tobacco products. The term “tobacco product” is defined quite broadly in FDCA Section 201(rr) as “any product made or derived from tobacco that is intended for human consumption, including any component, part, or accessory of a tobacco product (except for raw materials other than tobacco used in manufacturing a component, part, or accessory of a tobacco product)”. However, the law only gave FDA immediate authority, through its new Center for Tobacco Products (CTP), over specific types of tobacco products (e.g., cigarettes, cigarette tobacco, roll-your-own tobacco, and smokeless tobacco), while permitting the agency to “deem” other tobacco products (e.g., cigars, pipe tobacco, hookah (waterpipe) tobacco, e-cigarettes or e-liquid that contain tobacco-derived nicotine, etc.) to be regulated tobacco products using its rulemaking authority. 

On 25 April 2014, FDA issued a Notice of Proposed of Rulemaking for its “Deeming Regulation” that would extend the CTP’s tobacco authority to cover additional products that meet the tobacco product definition [25]. The final rule was published on 10 May 2016 and became effective on 8 August 2016 (81 Fed. Reg. 28973, 10 May 2016). Now, newly covered tobacco products are subject to virtually the same regulatory requirements as the currently regulated products including, among other things, adulteration and misbranding prohibitions, U.S. manufacturing establishment registration, product listing, ingredient reporting, testing for Harmful and Potentially Harmful Constituents, ban on unauthorized modified-risk claims and, most significantly, premarket authorization requirements. The Deeming Regulation also imposes minimum age restrictions on sales and requirements to include health warnings on product packages and in advertisements. 

At EU level, the Tobacco Product Directive [22] includes general provisions specific to “tobacco products” and “electronic cigarettes and herbal products for smoking”, which encompasses e-vapor products. E-cigarettes are defined in TPD Article 2(16) as “a product that can be used for consumption of nicotine-containing vapor via a mouth piece, or any component of that product, including a cartridge, a tank and the device without cartridge or tank” and that “can be disposable or refillable by means of a refill container and a tank, or rechargeable with single use cartridges”.

The term “refill container” is defined in TPD Article 2(17) as “a receptacle that contains a nicotine-containing liquid, which can be used to refill an electronic cigarette”, which captures the liquid-holding “tank” portion of e-cigarette devices, as well as e-liquid bottles sold directly to consumers. Generally, zero-nicotine e-liquids are regarded as outside the scope of the TPD. However, EU Member State law transposing the TPD may be broader in scope, and contain provisions and requirements relating to zero-nicotine e-liquids.

Electronic cigarettes that do not make medicinal claims and contain nicotine at a level below 20 mg/mL are regulated under Article 20 of the TPD. This Article allows the marketing of e-cigarettes, subject to certain nicotine content, volume and dose, quality and performance requirements, and an obligation to notify the relevant Member State before marketing, plus a number of other conditions and restrictions. 

Even though the threat of medicines regulation appears removed for products that satisfy these conditions, the regime largely mirrors medicines regulation without the benefits, namely, without the ability to advertise the product and be prescribed by the doctors as a smoking cessation tool. 

Furthermore, the TPD was inspired by a too-narrow interpretation of the precautionary principle [26]. An example is provided by Article n. 43, which literally states “electronic cigarette can develop into a gateway to nicotine addiction and ultimately traditional tobacco consumption, as they mimic and normalize the action of smoking. For this reason it is appropriate to adopt a restrictive approach to advertising electronic cigarettes and refill containers”. Some authors argue that there is an increased relative risk of smoking associated to EC use, especially among the youngest [27,28,29]. But this trend has recently reversed [30] and many of these young people were experimenting and certainly were not using e-cigarettes regularly. Moreover, experimentation with e-cigarettes is now associated with an accelerated rate of decline in smoking among youths [30]. This is true not only in the young population. As stated by the Royal College of Physicians in 2016, “to date, there is no evidence that any of these processes is occurring to any significant degree in the UK, where e-cigarette use is highly prevalent. Rather, the available evidence to date indicates that e-cigarettes are being used almost exclusively as a less harmful alternatives tobacco smoking” [31].

The incorrect identification of vaping and traditional smoking has been remarked also by the Italian Constitutional Court Judgment n. 83/2015, which rejected the authorization regime and tax norms applicable in the year 2014, concerning the sale of electronic cigarettes. In this judgment, the constitutional court highlighted the manifest unreasonableness that would derive from the contested norms, which introduced, without any scientific rationale, an analogous treatment for tobacco products and products suitable to replace the tobacco consumption, regardless of whether they contain nicotine or not [32]. Nonetheless, the Italian legislator did not take into account these considerations, and implemented the TPD with Italian Legislative Decree n. 6 of 2016. An overview of the key similarities and differences of the two regulatory frameworks (US FDA deeming regulation vs. EU TPD) has been condensed in Table 1 below.

## 2. Principles to Guide Policy Makers

When regulating e-cigarettes, evidence-based arguments should be taken into account in order to address all eventual concerns while minimizing potential unintended consequences of ill-informed regulation. Some principles and solutions that should guide policy makers towards a more reasonable regulation of these products include: the concept of tobacco harm reduction, the importance of relative risk and risk continuum, the possibility of de-normalization of smoking, the availability of low-risk products, the importance of a proportionate taxation and of consumer perspectives, and the value of a reassessment of the role of non-tobacco flavors. We will address each one of these principles, taking into account concerns, eventual misinterpretation of scientific data and proposing possible solutions. We will finally show the usefulness of a Risk Assessment Matrix to evaluate the probability that a risk associated to ECs use will occur and estimate the impact of such a risk on public health, so to finally decide if a contingency plan has to be implemented or if it would be better to keep the status quo.

### 2.1. Tobacco Harm Reduction

The harm of tobacco smoking at the individual and societal levels is well established. Cigarette smoking is the leading cause of avoidable premature mortality in the world and quitting is known to reduce the risk of fatal diseases such as lung cancer, acute coronary artery disease, strokes, end-stage chronic obstructive pulmonary disease and other cancers [35]. The World Health Organization Framework Convention on Tobacco Control advises that one of the key actions to reduce health burdens associated with combustible tobacco use is to encourage abstinence among smokers [36].

What seems hard for the public to appreciate, and for many scientists and regulatory agencies to accept, is that the problem of tobacco smoking is not nicotine, but the thousands of chemicals, tar and other toxic substances identified in tobacco smoke. It is not nicotine that is causing smoking-related diseases; nicotine is not a carcinogen and does not promote cardiovascular disease or obstructive lung diseases [37,38,39]. It is anticipated that any product delivering nicotine without involving combustion, such as e-cigarettes, would confer a very significantly lower risk compared with conventional cigarettes and other combustible sources of nicotine. 

In addition, the notion that nicotine may have significant positive effects is largely neglected. Akin to caffeine and several other psychoactive substances, it is not surprising that nicotine can improve several cognitive aspects of the individual including memory, level of attention, alert response and response time [40]. This does not mean that nicotine in tobacco cigarettes is entirely risk-free. The addictive potential of nicotine is well documented [41], and the Tobacco Advisory Group of the Royal College of Physicians [42] acknowledges that the development of addiction includes modifications in behaviour together with changes in brain structure and function that impair the ability to achieve and sustain abstinence. Many smokers experience long-term, perhaps lifelong, disruption in their mood and/or cognitive ability following smoking cessation. Consequently, they may never be able to give up all use of nicotine and may require long-term treatment, support, or nicotine maintenance to enable them to sustain abstinence from smoking. For this kind of smokers, tobacco harm reduction (THR), the substitution of low-risk nicotine products for cigarette smoking, could be a realistic compromise likely to offer substantial public health benefits [43].

The concept of harm reduction has been applied with success to several risky human behaviours, including drug use, automotive design, food processing, alcohol use, and sexual practices. The THR philosophy intends to be a third option between a high-risk lifestyle choice and its complete abstinence/prohibition, when the elimination of the smoking behaviour is not achievable. Indeed, although potentially millions of lives could be saved, the focus of most health regulatory authorities and tobacco control policies has been to encourage complete and simultaneous abstinence of smoking, tobacco use and nicotine among smokers, regardless of relative risk. For existing smokers, advice by the health authorities is almost exclusively focused on either quitting unassisted or with the use traditional nicotine replacement therapies (NRTs) supported by the medical community, such as the nicotine patch and gum, despite the well documented long-term effectiveness of those products. This approach, however, ignores the reality that many smokers cannot or do not want to achieve this goal, and for these smokers the alternative of reducing the risk of smoking-related diseases while continuing to take clean nicotine is the only realistic option [44].

This raises a key point about the goal to be pursued through any regulatory regime. Is the objective of such policies the greatest reduction in nicotine use or the greatest reduction in the deaths and illnesses caused by the way nicotine is delivered? This is important given that while there are risks associated with nicotine itself there are also benefits, and the overwhelming burden of disease associated with its use is due to combustion-based delivery being an incredibly dangerous delivery system. If the regulatory goal is one of public health a trade-off between a potentially higher rate of nicotine use and a dramatically lower health toll is good policy, and regulations should acknowledge it. 

With this in mind, pharmaceutical and tobacco industries have developed and marketed several smoke-free nicotine containing products, such as NRTs (i.e., nicotine replacement therapy, in form of gums, patches, sprays, tablets), and smokeless and/or dissolvable nicotine-containing products (such as snus, dissolvable tobacco, moist snuff, and dissolvable orbs, strips, and sticks). However, they all share an important shortcoming. Currently, these products do not compensate for relevant factors associated with the smoking experience (e.g., smoking rituals, sensorial sensation, etc.).

One researcher’s vision [45] of the ideal replacement for tobacco cigarettes, written nearly two decades ago, sounds remarkably like today’s e-cigarette products:

“If people have difficulty overcoming both nicotine dependence and long-term habit change, then surely the solution is to help them avoid most of the health risks with only a minimal alteration in their nicotine-seeking habits. This implies a nicotine replacement device, which looks like a cigarette and delivers cigarette-like boli of nicotine, but does not deliver the tar and carbon monoxide that cause the vast majority of smoking-related disease. The development and promotion of such a product (and its eventual replacement of tobacco) could have massive beneficial public health implications lasting into the 21st century”.

E-cigarettes could be the most attractive alternative to tobacco cigarettes because they share many behavioural and sensorial aspects of conventional smoking, including the hand-to-mouth repetitive motion and the visual cue of a smoke-like vapour [1]. In addition, they have a good THR potential, as discussed in detail in two recent review articles [9,43], not only for those smokers for whom current cessation programmes have had only limited success, but also for those inveterate smokers who consider their tobacco use a recreational habit they wish to maintain in a more benign form, rather than a problem to be medically treated.

Moreover, research shows that even in vulnerable populations of smokers with comorbidities, the e-cigarette may serve as a tool to reduce smoking and also to have an improvement in the comorbidity outcome. A retrospective study, for example, demonstrated improvements in asthma control, airway hyper responsiveness (AHR) and pulmonary function in asthmatic smokers who quit or dramatically reduced their cigarette consumption by switching to e-cigarettes [46]. Therefore, e-cigarette use in asthmatic smokers unable or unwilling to quit could be a valid and safer alternative approach to harm-reversal (i.e., specific reversal of asthma-related outcomes) and, in general, to harm-reduction (i.e., overall reduction of smoke-related diseases). 

Obviously, not all e-cigarettes should be treated equally; in particular, the modest effect and appeal with existing entry models (i.e., cigalike) cannot be compared to the more satisfying smoking-like experience of higher quality products. In a 2-year prospective observational study, it was shown that while some e-cigarette users who tried early generation cigalike products eventually relapsed back to tobacco smoking, many others graduated to adopting more rewarding advanced models [47]. More recently, in a 6-month pilot study, we have shown high retention and success rates with second-generation e-cigarettes (greater than those of “cigalikes”) in smokers not intending to quit [48].

### 2.2. Risk Continuum: Nicotine Concentration, Toxicants and Other Health Risks

The alternative of reducing the risk of smoking-related diseases while continuing to take clean nicotine becomes a realistic option with the current availability of a wide range of nicotine-containing products. However, not all alternative nicotine-containing products should be treated equal in term of health risk. Given that products’ impact on human health vary extensively according to the presence of toxins or potentially hazardous constituents, it is important for policy makers, health professionals and consumers to consider the relative level of harms of nicotine-containing products across a continuum, the so-called “risk continuum” [42].

In 2013, an international expert panel convened by the Independent Scientific Committee on Drugs developed a multi-criteria decision analysis model of the relative importance of different types of harm related to the use of nicotine-containing products [49]. After having selected 12 products and 14 harm criteria, the group scored all the products on each criterion for their average harm using a scale with 100 defined as the most harmful product, and a score of zero defined as no harm. This decision conferencing process confirmed tobacco cigarettes as the most harmful product (score of 100), with small cigars in second place (score of 64). Patches, gums and inhalers containing nicotine were not completely risk-free, but the risk was irrelevant when compared to tobacco cigarettes. The e-cigarettes were also placed in this low-risk group with a total risk index of 4, with the risk of perpetuating the smoker’s nicotine addiction over time mainly accounting for this score. This study clearly illustrates a risk continuum with conventional cigarettes at one end and NRT products and e-cigarettes at the other end. Of note, differences among products are substantial and if policy actions could help to switch use away from cigarettes and other combustible tobacco products to clean nicotine-containing products, massive public health gains would occur.

More recently, some critics of e-cigarettes have expressed concerns that consumers don’t know how much nicotine they are getting from these products and may become exposed to the risk of nicotine overdose. Because of these concerns, recent policy actions in EU have been designed to apply tight limits on nicotine levels in e-cigarettes. However, these decisions were not based on scientific evidence. Compared to conventional cigarettes, current e-cigarette models are not very efficient nicotine delivery systems. Generally, the amount of nicotine delivered from an equal number of puffs is much lower compared with smoking a combusted cigarette [50]. This is in agreement with the observation from other researchers that nicotine amount consumed [51] and absorbed [52,53] during e-cigarette use is quite low and with recent evidence suggesting that nicotine cannot be delivered as fast and as effectively from e-cigarettes compared to tobacco cigarettes [54]. 

Moreover, the risk of nicotine overdose or intoxication is unlikely to occur with vaping given that users will self-titrate their nicotine intake in a similar way to tobacco cigarettes [55]. Feelings of dizziness or nausea are signs of excessive intake of nicotine as compared to feeling of tiredness, numbness and confusion, which are signs of low circulating levels of nicotine. Consequently, smokers self-regulate nicotine intake to meet their psycho-physical needs [56,57,58]. It is rare that the average vaper or the dual user experience overdose symptoms (e.g., progressive agitation, nausea, vomiting, tachypnea—rapid breathing—, tremors and loss of consciousness). Last but not least, many e-cigarette users progressively reduce nicotine strength over time [8], and some have shown to abstain from smoking with zero-nicotine liquid [43].

A more accurate positioning of the e-cigarette across the “risk continuum” very much depends on the presence of potentially hazardous emissions and their impact on human health. The increasing knowledge about the presence of toxicants, metals, and chemicals in e-cigarette vapours has often been misrepresented or misinterpreted by health authorities and tobacco regulators in such a way that the potential for harmful consequences of e-cigarette use has been greatly exaggerated, thus generating out-of-proportion concerns and scares among health professionals and consumers.

Although the vast majority of the >7000 chemicals present in tobacco smoke [59] are completely absent from e-cigarettes, some toxic chemicals have been identified in the e-cigarette vapour [9]. However, their levels are substantially lower than found in tobacco smoke, and in some cases—such as nitrosamines—are comparable with the levels found in pharmaceutical nicotine products [60]. Moreover, given that e-cigarettes have several metal parts in direct contact with the e-liquid, trace level contamination with metals in the vapour is not unusual [61,62]. Overall, current data on the chemistry of aerosols and the liquids of e-cigarettes show that there is no evidence that vaping produces inhalable exposure to contaminants in the aerosol that would warrant health concerns [63].

In spite of the on-going commitment of many in the e-cigarette industry towards quality and safety improvements, some residual risk remains unavoidable for the time being. More research is needed in several areas, such as atomizer design and materials to further reduce potentially toxic emissions and improve nicotine delivery, and liquid ingredients to determine the relative risk of the variety of compounds (mostly flavourings) inhaled. From this point of view, it would be desirable to provide consumers with more information on the correct use and maintenance of the e-cigarette (in order to reduce any potential residual risk deriving from a misuse of the device), as it happens for any other commercial product destined to retail consumers.

If appropriate product safety and quality standards are introduced which can give regulators comfort on the safety and quality of the e-cigarette category, they will be more likely to introduce less restrictive regulations, which in turn would promote the harm reduction agenda. Moreover, suitable product standards would give consumers the necessary assurance, thereby encouraging an increase in the current rate at which smokers are switching to e-cigarettes. Last but not least, any residual risk associated with e-cigarette use is probably trivial compared with the devastating consequences of smoking. Moreover, e-cigarettes are recommended almost exclusively to smokers or former smokers as a substitute for conventional cigarettes or to prevent smoking relapse; thus, any risk should be estimated relative to the risk of continuing or relapsing back to smoking.

### 2.3. Population Health Matters: Gateway to Smoking or Out of Smoking?

Some public health professionals have expressed concern about e-cigarette use becoming a gateway to smoking or becoming a new form of addiction among youngsters or never-smokers. 

Most of the research to date has shown that e-cigarette use by adolescents is virtually non-existent unless they are already smokers. In a survey conducted in 1719 adolescents, first ever use of e-cigarettes in the past 30 days was reported in only five non-smokers [64]. Of note, “first ever use of e-cigarettes in the past 30 days” does not necessarily imply daily e-cigarette use, as it is also likely to include experimentation. Nor does it mean that this experimentation was with an e-cigarette containing nicotine. In the same survey, the prevalence of e-cigarette use was 2.3% in smoking adolescents. Data from the Centers for Disease Control National Youth Tobacco Survey [65] reported doubling in e-cigarette experimentation by 13–18 year old students from 1.1% in 2011 to 2.1% in 2012; however, 90.6% of them were smokers. Others were smokeless tobacco users. From the whole population, only 0.5% was constituted of non-smokers experimenting with e-cigarettes. Once again, participants were asked about ever experimenting with an e-cigarette in the past 30 days, not regular or daily e-cigarette use, or whether they were using a nicotine-containing product. A survey of more than 75,000 students in South Korea found that 12.6% of them were daily smokers, and only 0.6% of non-smokers had used e-cigarettes in the past 30 days [66]. 

Current analyses show virtually no never-smokers are using these products. Indeed, e-cigarette trial and use were found predominantly among current smokers (daily and nondaily), with some ex-smokers reporting regular use of e-cigarettes, but only 0.1% of never-smokers reporting current use [67]. Likewise, the MHRA found that less than 1% of e-cigarette users in the UK are never-smokers [68]. A similar conclusion was reached by a longitudinal survey in Swiss men [69]. Among current smokers, 9.3% (222/2376) had used e-cigarettes, and 12.2% of those (27/222), were daily users. Among former smokers, 1.6% (22/1343) had used e-cigarettes and of those, 13.6% (3/22) used them daily. Among never-smokers, 0.4% (5/1362) had tried e-cigarettes and none were using them regularly. 

Although a recent survey conducted in the US seems to show that e-cigarette use is increasing rapidly among adolescents [70], it must be noted that in a cross-sectional study the observed relationship between e-cigarette use and higher and more sustained levels of smoking does not imply causation. Moreover, such study does not take into account other population characteristics, which may play a crucial role when determining potential causation [71].

It must be said that a cross-sectional survey on 1500 10–11-year-old children in Wales [72] found that approximately 6% of children, including 5% of never smokers, reported having used an e-cigarette. By comparison to children whose parents neither smoked nor used e-cigarettes, children were most likely to have used an e-cigarette if parents used both tobacco and e-cigarettes (OR = 3.40; 95% CI 1.73–6.69). Nonetheless, we have to take into account that these children used an e-cigarette regardless of the law prohibiting sales to minors, and that minors tend to model parents behaviour (alcohol and smoking habits modelling parents are also widespread among adolescents). 

Although the theoretical concern about e-cigarette use becoming a gateway to smoking or becoming a new form of addiction has been used to warn about a new nicotine/tobacco epidemic, in actual fact the evidence shows that e-cigarettes are being used almost exclusively by smokers as a gateway away from smoking [73] and e-cigarette experimentation among young people has been consistently associated with acceleration in the rate of decline in youth smoking in the U.S. [30]. Nonetheless, we acknowledge that establishing a gateway effect to smoking as well as away from smoking is challenging, because any meaningful analysis must deal with simultaneity and confounding by common cause and in fact none of the studies purported to show a gateway effect from tobacco harm reduction products actually does so [74].

Another common concern is that widespread use of e-cigarettes may undermine tobacco control or “re-normalise” smoking because by diverting smokers from quitting, these products may lead to an increase in smoking prevalence, or at least a slowing down of the rate of decline. Yet, current evidence points in the opposite direction, with the rise in prevalence of e-cigarette use being associated with an increase in smoking cessation rates and a continued fall in smoking prevalence [75,76].

Others are concerned that e-cigarette use may “re-normalise” smoking by visible displays of smoking-like behaviour. There is no basis to believe that this association is real. In contrast, seeing other people using e-cigarettes may prompt many smokers to switch to a much less harmful product, thus de-normalising *de facto* tobacco smoking [26].

### 2.4. Emerging Regulatory Challenges: Consumer Perspectives, Restrictions and Flavors

The main reason for regulation of e-cigarettes is to ensure that consumers are protected. However, consumers’ perspectives have been largely overlooked. For consumers, safety is a concern, but is secondary in view of the hazards of the product (i.e., tobacco cigarette) being replaced. Most consumers would be content with regulations that helped to ensure product consistency and prevent contamination, but see no need to apply the strict regulations used for pharmaceutical or tobacco products that would lead to reduced product availability and unnecessary increases in the price of e-cigarettes [77]. In a pilot longitudinal study conducted in Atlanta (GA, USA) in 2013, to assess the attitude and perceptions about the EC in regular smokers who were first-time e-cigarette purchasers, the 97.2% of participants believed that ECs have fewer health risks than regular cigarettes, should be allowed where smoking is not, and have been shown to help smokers quit and to reduce cigarette consumption. More interesting, only 27.8% believed that the FDA should regulate e-cigarettes. According to the authors, this may be related to little understanding of the role of the FDA in protecting and informing consumers. The other consumers may generally reject high levels of regulation and assume that regulation will impact access to e-cigarettes [78].

Our experience suggests that many former smokers who transitioned to an e-cigarette believe that the main goal for regulators should be to keep e-cigarettes available and acceptable as a cigarette replacement. Excessive and ill-conceived regulation will conflict with these basic requirements; it will marginalise e-cigarettes by making them unattractive to smokers and less competitively priced compared with tobacco products [4].

Another challenge faced by regulators is to prioritize novel potential questions of public health raised by e-cigarettes, without losing sight of the fact that the fall-back status quo translates into a continuation of the epidemic of diseases caused by smoking. This requires careful reframing of harm reduction strategies in a comprehensive manner, leveraging the harm reduction potential of currently available low-risk nicotine-containing products. Simply responding to a potential risk without gathering sufficient data often results in unintended consequences that can negatively impact public health. Here we will list a few examples.

The restrictions that some in the tobacco control community wish to impose on e-cigarettes range from outright bans on all sales to limitations on product features, all of which would appear to do more harm than good. One city in California passed legislation prohibiting the approval of licenses for stores to sell e-cigarettes [79]. Lawmakers have proposed imposing special taxes on e-cigarettes and halting Internet sales [80]. Some localities have proposed defining e-cigarettes as conventional cigarettes, which would bring into play a number of controls, including restrictions on where products may be sold, and, in some cases, requirements to purchase products for resale from tobacco wholesalers. These measures would reduce the availability of e-cigarettes as a low-risk substitute for smoking and would have the unintended consequences of perpetuating smoking. In fact, anything that continues to give tobacco cigarettes an advantage in the marketplace will have negative implications for public health and consumer rights. 

Another way to impose prohibitionist restrictions on low-risk products is through taxation. U.S. Supreme Court Chief Justice John Marshall declared, “The power to tax involves the power to destroy” [81]. In the case of tobacco, tax policy has been, to date, arguably the single most powerful tool in reducing consumption, a point made by the WHO in its 1996 World No Tobacco Day documents and discussed in reports of the World Bank [82]. Reducing the affordability of cigarettes reduces onset of smoking by young people, and encourages cessation and smoking reduction among existing smokers. But these measures also have unintended consequences in that the higher prices for legitimate product can lead smokers to contraband sources of supply, find cheaper legal alternatives such as hand-rolled cigarettes, or otherwise engage in behaviours that do not improve health [32].

Since consumers move between competing alternatives based on price, we also have to consider cross elasticity when looking at taxation policies, because price differentials incentivize consumers to change behaviour. To date, with few exceptions—for example, a higher alcohol content beverage is typically taxed at higher rates than a lower alcohol content beverage to discourage excess alcohol consumption—this very powerful tool has played little role in health-focused policy making in this area. This is in part because of a lack of acceptable lower-risk alternatives for smokers in most countries. But this situation is now changing, and there is increasing awareness that cross elasticity can very effectively move smokers from cigarette use to the use of non-combustion products, as seen in a recent report from Citi Research [83]. A report from the National Center for Policy Analysis points out, “if the rate of taxation is equal to or higher than cigarettes, the state is penalizing users who switch to a less risky product” [84]. The greater the differentiation in relative risks between products, the greater the basis for a differentiation in taxation. Excise taxes are not levied on NRT for very good reasons, and similar thinking could be warranted for e-cigarettes.

Tobacco taxation policy is not the only anti-tobacco measure that regulators are willing to apply to low-risk products. More recently, flavours in e-liquids have become a cause for concern, with health regulators proposing their outright ban. In the U.S., characterizing flavours in conventional cigarettes were banned by FDA in 2009, and some groups believe that the same should apply to non-combusted products. However, there is a definite paucity of research backing their proposal, and little consideration, if any, seems to be given to the consequences of failing to offer smokers more acceptable alternatives to their cigarettes. A press release from the Campaign for Tobacco-Free Kids states, “Electronic cigarettes have been marketed in youth-friendly candy and fruit flavors including bubblegum, cookies and cream, and cola” [85]. At first blush, banning flavours might seem like a way to discourage adolescents from trying e-cigarettes, but the question needs to be asked whether the flavours are what attract kids to try the products. Nicotine gum and lozenges are marketed to adult smokers. The original flavour of gum has been described as “peppery”, and many smokers found the taste and the sensation unpleasant [86]. Researchers reported, “because the taste of nicotine gum has impeded compliance with dosing recommendations, nicotine gum with improved taste (mint, orange) was developed and marketed” [87]. Today these products come in flavors such as Cinnamon Surge, Fruit Chill, and White Ice Mint. Despite these friendly sounding flavors, rarely does any nonsmoker—youth or adult—takes up use and becomes addicted to nicotine. At a given nicotine dose, some flavors in nicotine gums are better than others at reducing withdrawal symptoms (such as anxiety, dysphoria and tension). For example, it has been shown that vanilla and baked apple cardamom flavored gum resulted in lower levels of negative affect associated with nicotine withdrawal while peppermint flavored gum was not different from the plain gum [88]. This clearly suggests that flavors may play a major role in helping smokers quit.

In an effort that replaces possible “risks” with actual data, the UK organization Action on Smoking and Health conducted a survey of children ages 11–18 [89] and reported, “Among children who have heard of e-cigarettes, sustained use is rare and confined to children who currently or have previously smoked”. University of North Carolina Researchers surveyed 228 adolescent males, ages 11–19, and found that only two participants had previously tried e-cigarettes [90]. They also found that willingness to try plain versus flavored varieties did not differ. Initially, most adult e-cigarette consumers start out using tobacco or menthol flavors. Many discussions in e-cigarette consumer web forums are about seeking advice on finding a flavour that matches their old brand of combusted cigarettes [91]. When that doesn’t work, many try a more pleasant flavour just as an experiment, and often end up preferring non-tobacco flavors so much that the taste of tobacco becomes abhorrent [92]. The Consumer Advocates for Smoke-free Alternatives Association survey [10] found that 70.1% of the participants use fruit, beverage, or candy flavored liquid at least “Occasionally”, with 16.3%, 18.1%, and 17.5% using such flavors “Regularly”, “Often”, or “Always”, respectively. What’s more important is that 73% indicate that the availability of these flavors are influential in their continued use of the products, which may help to prevent relapses to smoking. 

Recent research showed that some chemicals were found in a large proportion of sweet-flavored liquids, at levels that were higher than the strictest safety limits—but significantly lower compared to smoking [93]. But any presence of potentially dangerous chemicals in e-cigarette liquids represents an avoidable risk, dealt with simply by adopting proper safety measures by manufacturers and flavouring suppliers to eliminate these hazards from the products, without necessarily limiting the availability of sweet flavors [4].

## 3. Recommendations: Proposal for a Risk Assessment Matrix

Taking action to limit hypothetical risks can have unintended consequences that work against achieving the ultimate goal of saving lives from the world first preventable cause of death, tobacco smoking. Therefore, it is important to first evaluate the probability and impact of each risk against the mitigation strategy before deciding to implement a contingency plan.

We would suggest to the policy makers a way to determine, first, the probability that a risk will occur and then the impact that this would have, through a Risk Assessment plan.

Table 2 below illustrates how the risks perceived and expressed by the concerns of policy makers and health organizations could be evaluated according to a Risk Assessment Matrix, which we adapted from the sample Risk Management plan provided by the U.S. Department of Health & Human Services in the Public Health Emergency toolkit [94].

Take, for example, the potential risk of allowing unregulated liquids and devices to be sold. Specifically, there might be a risk of poisoning if toxic chemicals were to be added to the liquids that are vaporized during the operation of ECs. The Impact of this could be Catastrophic (A) if death or disability results. But it is important to determine the probability by considering the frequency with which this has been occurring. Since it has never been reported, the Probability would have a value of 1 (Improbable), resulting in a Risk Level of 1A, Medium Low, for consumers being poisoned by contaminated liquids.

Another specific example would be the danger of nicotine overdose for EC users. For liquids that contain no nicotine, the Probability would be Improbable (1), and the Impact would of course be negligible (E), resulting in a Risk Level of 1E (Low). For liquids that do contain nicotine, how serious is a nicotine overdose likely to be? We can look at historical information for other products that deliver nicotine such as smoking conventional cigarettes or chewing nicotine gum. No deaths have ever been reported for normal use of such products. The only known death from nicotine patches occurred when a man covered his body with patches--a probable suicide. The only known death from EC liquid that contains nicotine occurred in a man who committed suicide by injecting the liquid directly into his veins. Again, the Probability would be a 1, and while the Impact for an individual could be Catastrophic most EC consumers who take in too much nicotine experience temporary effects such as nausea. Thus the overall impact on public health would be Negligible, resulting in a Risk Level of 1E (low). 

Even if it could be subjective, the Risk Level should be quantified whenever possible and can be used as the basis for determining the degree to which actions should be taken to mitigate the risk. The next step of regulators should be to identify the potential actions that might reduce the probability or the impact of a High Risk event. All potential solutions should be explored, including making no change at all, and should include a determination of the possible unintended consequences of implementing each solution [13]. Ideally, the selected solution will be the one that has the greatest potential for reducing morbidity and mortality. Once solutions have been implemented, regulators should monitor the results and adjust or eliminate regulations that are not having a beneficial effect.

If no surveillance information is available, research can be conducted to provide estimates of the probability that a perceived risk will occur and the impact of failing to address the risk through regulatory action. If the available data is insufficient or imprecise and doesn’t allow regulators to determine with certainty the existence or extent of the risk feared, but there is the likelihood of real harm to public health if foreseeable risks materialize, the precautionary principle justifies the adoption of restrictive measures, provided that they are objective and non-discriminatory, and take into account the risk of continued smoking. The key element of the principle is that it allows anticipatory action in the absence of scientific certainty. However, the process of applying the principle must be “open, informed, and democratic, and must include potentially affected parties. It must also involve an examination of the full range of alternatives, including no action” [95].

So far, most regulatory bodies have failed to include the parties most deeply affected by the regulation of e-cigarettes: consumers. Regulators have also failed to examine the full range of alternatives, including taking into account the health risks of maintaining the status quo, continued smoking [26]. To give a concrete example, if the concerns about population effects cannot be easily managed, it is due to the fact that it is difficult to know for sure in advance how e-cigarettes will affect uptake by never smokers or an eventual re-normalization of smoking. In this case, the precautionary principle would require the regulator to look at uncertainty about population effects, but it should be done symmetrically, namely, recognizing that regulatory actions such as a ban could have a cost in the form of benefits foregone.

## 4. Conclusions

Involving consumers in the regulatory process, taking into account the best available science, identifying risks and assess their occurrence and impact is the best strategy for policy makers to determine which concerns represent real problems and point out potential unintended consequences of any proposed actions that they might overlook.

Future regulatory measures about ECs should primarily address safety and quality standards, child-proof caps on liquid containers, verification of accurate nicotine levels and product ingredients, complete and accurate package labelling and safety warnings, and post-marketing surveillance [26,96]. This is the basis of a proportionate legal framework that could maximize the impact of these alternative products as a low-risk replacement for smoking conventional cigarettes, while minimizing the risks.

## Figures and Tables

**Table 1:** FDA vs. EU e-cigarette regulation: key elements for comparison.

	FDA Deeming Regulation	EU Tobacco Product Directive (TPD)
Effective as of:	8 August 2016	20 May 2016
General Statement	Regulations stated in general terms without specification to manufacturers as to what will and will not be acceptable	Regulations specific as to what manufacturers must comply with
E-cigarette regulated as:	Tobacco product. In the final Deeming Rule, FDA stated that all Electronic Nicotine Delivery Systems (ENDS) are subject to the Tobacco Control Act.	Tobacco product. Generally, zero-nicotine e-liquids are regarded as outside the scope of the TPD. However, EU Member State may contain provisions relating to zero-nicotine e-liquids. The TPD excludes e-cigarettes and e-liquids regulated as medicinal products or medical devices.
What local governments can do?	Cities and States can regulate age restrictions on sale above age 18, places where e-cigarettes are not allowed and taxation.	Member States can regulate further without going contrary to supranational framework. For example, they are responsible for adopting rules on flavors and age limits and can ban cross-border distance sales
Specific requirements	The Tobacco Control Act (TCA) requires that the marketing of a new tobacco product is appropriate for the protection of the public health, with respect to the risks and benefits to the population as a whole, including users and nonusers of the tobacco product. E-cigarettes and e-liquids are subject to the Tobacco Control Act requirements, including U.S. manufacturing establishment registration and product listing, ingredient reporting, health document submissions, testing for harmful and potentially harmful constituents (HPHCs), nicotine addiction warning requirements, age restrictions, prohibition of free samples, and premarket authorization. The TCA requires that all tobacco products not on the market as of 15 February 2007 submit a Premarket Tobacco Product Appication (PMTA) within two years of 8 August 2016 to remain on the market. All e-cigarettes products now on the market must meet this application. E-cigarettes companies that submit PMTAs by this deadline can continue marketing their products for an additional 12 months until 8 August 2019. If FDA is unable to complete its review during this 12-month period, the product will have to be removed from the market until FDA completes its review. Section 911 of the TCA specifies that no product can claim lower risk than any other product on the market (i.e., e-cigarettes cannot claim to be lower in risk than cigarettes) without submitting a Modified Risk Tobacco Product Application (MRTP). Requirements, application procedure and lack of guidance to manufacturers basically identical to the PMTA application. Section 911(a)(2)(C) of the TCA specifically prohibits any smokeless tobacco product from claiming lower risk just because it is smokeless.	Specific safety and quality requirements for every nicotine-containing EC (Article 20): - maximum nicotine concentrations (20 mg/mL) - maximum volumes for cartridges/tanks (2 mL) - e-liquids sold in dedicated containers not exceeding a volume of 10 mL - child-resistant and tampered proof e-cigarettes and liquid containers - anti leakage refilling - high-purity of the ingredients and no additives listed in Article 7(6) Under TPD Article 20(2), as of 20 May 2016, manufacturers and importers must submit in electronic format a notification to the competent authorities for any e-cigarettes or refill container product intended to be placed on the EU market. The notification must be submitted at least six months before the product is placed on the market. A common format has been established for the notification. The TPD has no regulations requiring application or otherwise addressing claims of reduced risk.
Process of product approval	PMTA required of all e-cigarette products [33] - a separate PMTA and/or MRTP application must be submitted for every combination of a device, flavor and strength of nicotine. - FDA must be notified in advance of any anticipated application with the understanding that additional requirements may be imposed by FDA staff at that time. - longer, expensive and more stringent process than EU notification. - a significant amount of information must be provided, which includes non-clinical data, and potentially animal studies and human clinical trials to satisfy the requirements above. - other deadlines for registration for manufacturers and importers, ingredient and health documents submissions, warning labels, and other requirements.	No new product application required for current or newly introduced products, but authorities must be notified [34] - standard process for every product, where specific information must be provided: manufacturer, components of the products, ingredients of the e-liquid, emissions, toxicological data, production process; - notification to the authorities for all products placed or intended to be placed on the market and annual report on sales volumes, consumer preferences and trends; - compliant products can be sold 6 months after notification, unless a particular problem arises.
Under-age sales	- FDA forbids the sale of e-cigarettes to those under the age of 18 years old. - Identity documents must be verified at retails, and a valid youth access check must be guaranteed also by online retailers. - Cities and states may impose higher age limits for purchase (usually 18 or 21).	TPD makes no explicit prohibition, but Member States are responsible for adopting age limits.
Marketing	- FDA does not currently prohibit online, TV, radio or print advertising of products other than cigarettes. - Free sampling is sharply restricted. - Any direct or indirect claim in an advertisement, web site or label that the product may be lower in risk or useful for smoking cessation will cause removal from the market - Health warnings not yet specified in law or regulation for e-cigarettes.	- The TPD does not harmonize rules on domestic sales arrangements or domestic advertising, or brand stretching, but notes that the presentation and advertising of those products should not lead to the promotion of tobacco consumption or give rise to confusion with tobacco products. Member States are encouraged to regulate such matters. - TPD does not prohibit free sampling. - Health warnings are mandatory.
Labelling and packaging	- Child-proof packaging required for packages of e-cigarette liquid intended for consumer use. - By 10 May 2018 labels for covered tobacco products that contain nicotine, including ECDS, need to include a nicotine addiction warning, with specific typographic requirements.	Any outside packaging of e-cigarettes and refill containers must have the following details: - List of all ingredients, information on the product’s nicotine content and delivery per dose, and a leaflet setting out instructions for use and information on adverse effects, risks, addictiveness and toxicity. - Promotional elements are not allowed on packaging, nor elements suggesting tobacco harm reduction, or economic or environmental advantages with respect to tobacco smoke. - A nicotine addiction warning must be included in the packaging, with specific typographic requirements.

**Table 2:** Risk Assessment Matrix.

Probability of Occurrences			Impact				
			Catastrophic	Critical	Moderate	Minor	Negligible
Definition	Meaning	Value	(A)	(B)	(C)	(D)	(E)
Frequent	Occurs frequently Will be continuously experienced unless action is taken to change events	**5**	**5A**	**5B**	**5C**	**5D**	**5E**
Likely	Occurs less frequently if corrective action is taken Documented through surveillance	**4**	**4A**	**4B**	**4C**	**4D**	**4E**
Occasional	Occurs sporadically Discovered through surveillance	**3**	**3A**	**3B**	**3C**	**3D**	**3E**
Seldom	Unlikely to occur Rarely, if ever, reported	**2**	**2A**	**2B**	**2C**	**2D**	**2E**
Improbable	Highly unlikely to occur Never previously reported	**1**	**1A**	**1B**	**1C**	**1D**	**1E**

Risk Levels: Risk is High for codes 5A, 5B, 5C, 4A, 4B, 3A (in red); Risk is Medium High for codes 5D, 5E, 4C, 3B, 3C, 2A, 2B (in orange); Risk is Medium Low for codes 4D, 4E, 3D, 2C, 1A, 1B (in yellow); Risk is Low for codes 3E, 2D, 2E, 1C, 1D, 1E (in blue).
